# Improved Iterative Decoding of Network-Channel Codes for Multiple-Access Relay Channel

**DOI:** 10.1155/2015/493767

**Published:** 2015-11-09

**Authors:** Saikat Majumder, Shrish Verma

**Affiliations:** Department of Electronics and Telecommunication, National Institute of Technology, Raipur, Chhattisgarh, India

## Abstract

Cooperative communication using relay nodes is one of the most effective means of exploiting space diversity for low cost nodes in wireless network. In cooperative communication, users, besides communicating their own information, also relay the information of other users. In this paper we investigate a scheme where cooperation is achieved using a common relay node which performs network coding to provide space diversity for two information nodes transmitting to a base station. We propose a scheme which uses Reed-Solomon error correcting code for encoding the information bit at the user nodes and convolutional code as network code, instead of XOR based network coding. Based on this encoder, we propose iterative soft decoding of joint network-channel code by treating it as a concatenated Reed-Solomon convolutional code. Simulation results show significant improvement in performance compared to existing scheme based on compound codes.

## 1. Introduction

The increasing popularity of ubiquitous computing has motivated the deployment of wireless communication devices that require ad hoc communication. One such application of pervasive computing is in wireless sensor networks (WSN), which is used to monitor the physical world parameters like temperature, radiation levels, image, video, and so forth. The WSN consists of large number of low cost wireless sensor nodes and each node itself has very limited computational capability. The nodes are further limited by limited power of transmitters. The main challenge of wireless sensor networks is to achieve proper balance between transmit/processing power and quality of service. However, such multiterminal systems are limited by impairments due to wireless channels, such as fading, and interference.

In the case of point to point communication systems, effect of fading is mitigated using multiple antennas at transmitter or receiver. Whereas wireless sensor nodes are too small to accommodate multiple antennas on a single terminal, several nodes can cooperate to obtain cooperative diversity [1]. In cooperative communication transmitting users use one another's antenna to realize the benefit of multiple antenna transmission. There are many cooperative strategies to achieve efficient node cooperation, such as amplify and forward [2–4], decode and forward [5], and coded cooperation [6, 7]. In amplify and forward protocol, relay nodes retransmit amplified versions of the signal received from source. Amplification coefficients at the relay nodes control the performance at the destination whereas in decode and forward relay nodes first decode the received symbol using hard decision and then transmit the reencoded signal to destination. Coded cooperation achieves space diversity by forwarding different segments of a channel code through different paths. Based on these basic schemes, various other improvements have been proposed in recent years. Zhou et al. [7] proposed a distributed joint source-channel coding technique that exploits source relay correlation. Throughput of the network can be increased further by using network coding at the relay nodes [8]. Network coding allows the packets to be mixed and combined at the relay nodes, instead of simple retransmission. In this paper we consider a scheme proposed in [9] employing multiple-access relay channel (MARC) where two user nodes obtain cooperation through a fixed relay node performing network coding. This system has advantage of diversity gain, as well as increase in system throughput. The benefit of network coding in MARC schemes has been demonstrated by other authors also. Authors in [10, 11] proposed schemes which combine the benefits of space-time codes and network coding for cooperative communication. The authors use simple XOR based network coding at the relay for obtaining diversity. They have demonstrated that combination of Alamouti space-time code and network coding outperforms system based only on Alamouti coded cooperation. Du and Zhang [12] investigated a cooperative strategy based on parity check network coding. Their study revealed that a successful design should employ the most effective extra check bits to make full use of information in relayed bits to help decode the message from two users. Fang et al. [13] proposed a joint network-channel coding scheme based on distributed turbo code for multiple-access relay channel, using decode and forward protocol. In [14] authors consider the problem of transmitting correlated binary sources over MARC. They proposed a joint source-channel-network decoding technique to fully exploit the correlation between sources.

In this paper we consider joint network-channel coding for multiple-access relay channel when the transmitting node employs Reed-Solomon (RS code) error correcting code and punctured recursive systematic convolutional code (RSCC) is used as network code. Encoding user information with RS code, in contrast to simply encoding with linear block code [9], is more relevant because of widespread prevalence of RS code in many existing standards. The main contribution of this paper is applying the concept of iterative soft decoder for concatenated codes [15] in the context of network-channel decoding in MARC setup. The proposed algorithm enables network code and channel decoders to exchange soft information iteratively and to achieve an improved performance compared to a hard decision iterative decoder. We apply extrinsic information obtained from network decoder to soft-input soft-output (SISO) decoder for RS code [16, 17] through an interleaver. The extrinsic output of SISO RS decoder is applied back to SISO network-channel decoder. SISO decoding of RS code allows its decoding beyond maximum-distance separable (MDS) capability, in contrast to popular approach of hard decision decoding of RS code. The convergence behaviour of the network-channel decoder is analyzed using EXIT (extrinsic information transfer) chart [18]. We demonstrate using bit error rate (BER) simulations that the proposed scheme performs better compared to existing design proposed in [9] and conventional XOR based network coding.

The proposed scheme is visualized for uplink transmission in wireless sensor networks, where sensed information is transmitted to a central base station. In such a scenario, one needs computationally less complex sensor nodes and relays but can bear more algorithmic complexity at the base station. The rest of the paper is organised as follows.  Section 2 describes the coding strategy and model of the system under consideration. In Section 3, the proposed iterative channel-network decoder is described, along with brief discussion of the component decoders.  Section 4 describes the EXIT chart analysis of the proposed decoding scheme. Bit error performance is obtained by simulation and compared to baseline scheme in Section 5. Finally, Section 6 is the conclusion and provides suggestions for future work.

## 2. System Model and Coding Strategy

**Figure 1:**
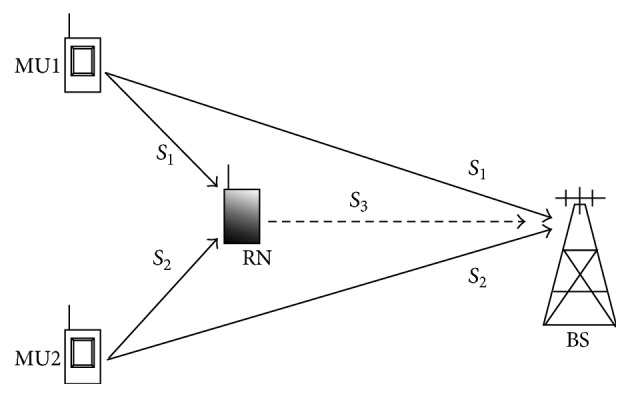
Multiple-access relay channel with two users transmitting channel coded information to a base station. Network coding operation is performed at the relay node.

We consider the scenario shown in Figure 1, with two mobile user nodes communicating information to a common base station in a MARC setup. Basically, the system consists of two mobile sources MU1 and MU2 transmitting binary data sequences *S*_1_ and *S*_2_, respectively, towards the relay node (RN) and a base station (BS). Both user nodes have a symmetric positioning with respect to RN and BS. Since radio terminals cannot transmit and receive simultaneously, the nodes are assumed to operate in half-duplex mode and are using orthogonal channels. The relay node decodes the intercepted packets, reencodes them, and performs network coding on the information received from both of the information nodes. Network coded packets are then forwarded to the base station in the second phase of transmission. Decoding is performed at the BS by combining the packets received from both the user and relay nodes. The detailed protocol showing transmitting nodes, their channel assignments, and the respective transmitted messages is shown in Table 1. In the next paragraphs we describe the encoding operations at source and relay in detail and decoder is discussed in subsequent sections.

### 2.1. Encoding at Mobile User Nodes

**Figure 2:**
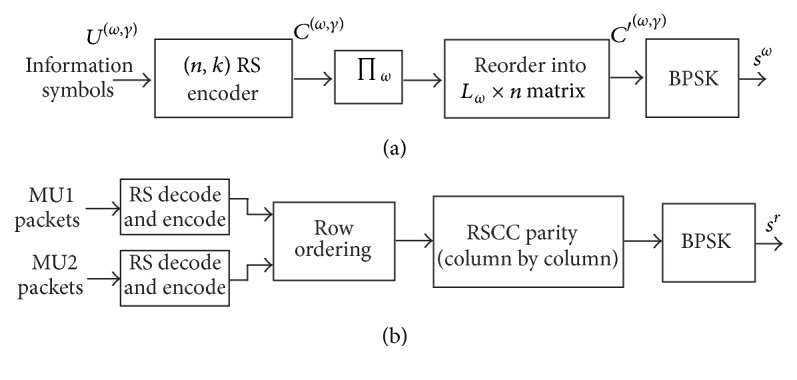
(a) Block diagram of encoder at mobile user nodes. (b) Network coding operation at relay node.

Let *F*_*q*_ denote the finite field of size *q*. It is assumed that *F*_*q*_ is an extension field of *F*_2_ as *q* = 2^*m*^. The message vector for an (*n*, *k*) RS code is given as *U* ^(*ω*, *p*)^ = [*U*_1_^(*ω*,*p*)^, *U*_2_^(*ω*,*p*)^,…, *U*_*k*_^(*ω*,*p*)^] ∈ *F*_*q*_^*k*^, where *n* and *k* are the length and dimension of the code, respectively, and *n* = *q* − 1. The superscript *ω* ∈ {1,2} denotes the mobile user MU and variable *p* denotes the *p*th codeword or packet. The *p*th RS code of source MU-*ω* is obtained as

(1) $$\begin{array}{c} C^{\left(\omega ,p\right)}=U^{\left(\omega ,p\right)}G=\left[\;C_{1}^{\left(\omega ,p\right)},C_{2}^{\left(\omega ,p\right)},\ldots ,C_{n}^{\left(\omega ,p\right)}\;\right]\in {\text{F}}_{q}^{n}, \end{array}$$

where **G** is the generator matrix of the RS code. In practice, the bits from each source are grouped into *m* bit symbols, *U*_*i*_^(*ω*,*p*)^ ∈ *F*_*q*_, for *i* = 1,…, *k*, and coded with (*n*, *k*) RS code. The encoding at MU nodes is shown in Figure 2(a). The RS code has dual functions; first, it is efficient against burst errors, since a sequence of *m* + 1 consecutive bit errors can affect at most two code symbols. Second, RS code aids in iterative joint network-channel decoding as discussed in the next section. At each source node, *L*_*ω*_ codewords are generated, grouped into a frame, and interleaved with Π_*ω*_. The stream is then formed into matrix of size *L*_*ω*_ × *n*, where each row forms a packet. Each packet is thus composed of randomized symbols from different RS codewords and is represented as *C* ^′(*ω*, *p*)^ = [*C*_1_^′(*ω*, *p*)^, *C*_2_^′(*ω*, *p*)^,…, *C*_*n*_^′(*ω*, *p*)^] ∈ *F*_*q*_^*n*^, where *p* now indicates packet number of each source. The symbol array *C* ^′(*ω*, *p*)^ is converted into bit array *c* ^′(*ω*, *p*)^ = [*c*_1_^′(*ω*, *p*)^, *c*_2_^′(*ω*, *p*)^,…, *c*_*mn*_^′(*ω*, *p*)^] ∈ *F*_2_^*mn*^. The translated bits are modulated and broadcast to BS and relay.

### 2.2. Encoding at Relay Node

**Figure 3:**
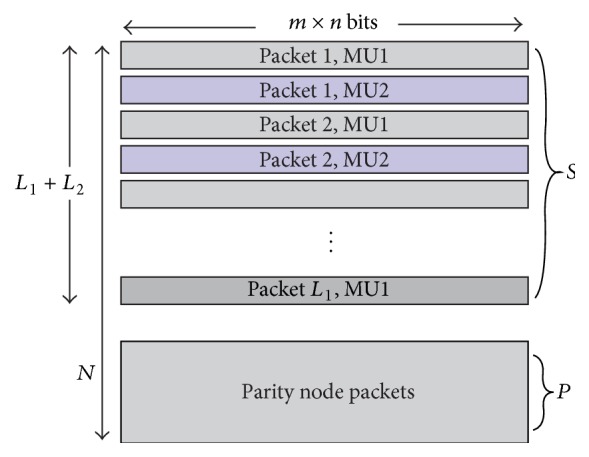
Data packets from two sources are ordered in alternate rows. The total number of rows from two sources is *L*_1_ + *L*_2_. Remaining rows of parity check bits are calculated and transmitted from the relay node.

The function of the relay node is performing network coding on the incoming packets from two sources. In case of simple XOR based network coding, one packet from source MU1 is linearly combined, using bit-by-bit XOR operation, with packet from source MU2 and the resulting bits are transmitted to the BS. The resulting network code at relay node has rate of 2/3.  Figure 2(b) shows the proposed encoding operation at the relay node. The relay node overhears transmission from both of the MU nodes during the first phase of transmission and decodes and reencodes them. The reencoded packets are ordered into matrix *S* of size *L* × *mn* bits, as shown in Figure 3, where *L* = *L*_1_ + *L*_2_. Here, we have the first *L* rows belonging to the cooperating users. From these packets, the remaining *N* − *L* rows are generated using network code and forwarded by the relay. In this research we use recursive systematic convolutional code (RSCC) as network code instead of XOR based network code. RSCC of rate *L*/*N* is applied on each column of *L* bits, from which *N* − *L* parity check bits are obtained. The parity check bits form matrix *P* of size *N* − *L* × *mn*. It is to be noted that relay retransmits only the parity part of convolutional code. Puncturing may be applied on the parity bits to attain necessary code rates. In this paper, every alternate row of the parity bits (*P* in Figure 3) is punctured to obtain code rate of 2/3. Any different puncturing pattern may be applied, but this may result in different performance. Network code is obtained from these parity check bits and each row is transmitted as packet to the BS. Thus each row encounters different channel and bits in a row suffer from the same amount of fading.

### 2.3. Channel Model

The wireless channel is assumed to be flat Rayleigh fading channel and the fading coefficient is constant for the duration of one packet transmission. Let the *p*th received packet at the BS from link *l* be denoted as *y*_*p*_^*l*^ = [*y*_*p*1_^*l*^, *y*_*p*2_^*l*^,…, *y*_*pn*_^*l*^], for *l* ∈ {1,2, *r*} and *p* = 1,…, *L*_*l*_. Thus, the received signal from link *l* and packet *p* is

(2) $$\begin{array}{c} y_{pi}^{l}=h_{pi}^{l}s_{pi}^{l}+z_{pi}^{l},\;i=1,2,\ldots ,mn, \end{array}$$

where *s*_*pi*_^*l*^ is the transmitted symbol of unit energy. The fading coefficients *h*_*pi*_^*l*^ are zero mean complex valued Gaussian random variables with Rayleigh-distributed envelope. The channel is assumed to experience slow Rayleigh fading such that fading coefficients are nearly constant over one codeword interval. Gaussian noise *z*_*pi*_^*l*^ experienced by the *i*th symbol of link *l* has double-sided power spectral density *N*_0_/2.

## 3. Iterative Network-Channel Decoder

**Figure 4:**
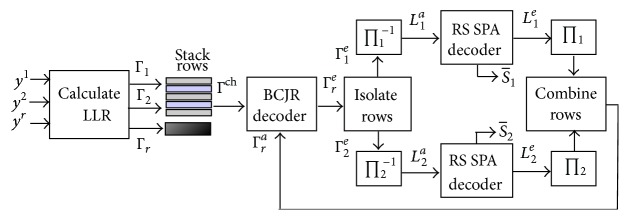
Block diagram of the proposed decoder.

The block diagram of the proposed iterative soft network-channel decoding algorithm is shown in Figure 4. At the receiver RSCC and RS codes can be considered a concatenated code structure and can be decoded iteratively [15]. The next stages consist of iterative soft decoding process in which MAP decoder is applied along the columns for soft-input soft-output (SISO) decoding of RSCC and adaptive belief propagation (ABP) algorithm [17] for SISO decoding of RS codes along rows. We denote the LLR of a received bit *s*_*pi*_^*l*^ of packet *p* from link *l* as *λ*_*l*_(*p*, *i*) and it is calculated as

(3) $$\begin{array}{c} \lambda_{l}\left(\;p,i\;\right)=\frac{\left({\left|y_{pi}^{l}+h_{pi}^{l}\right|}^{2}-{\left|y_{pi}^{l}-h_{pi}^{l}\right|}^{2}\right)}{N_{0}}. \end{array}$$

Channel state information *h*_*pi*_^*l*^ is assumed to be available at the base station. The LLRs of received packets are stacked over one another in the form of the matrix Γ^ch^ as given in Figure 3. Γ^ch^ consists of alternate rows of LLRs of packets from MU1 and MU2, while the last (*N* − *L*_1_ − *L*_2_) rows are LLR of packets received from RN. Γ_*l*_^*a*^, Γ_*l*_^*e*^, and Γ_*l*_^*p*^ denote the* a priori* LLR, the extrinsic LLR, and the* a posteriori* LLR, respectively, and are related as

(4) $$\begin{array}{c} \Gamma_{l}^{e}=\Gamma_{l}^{p}-\Gamma_{l}^{a}. \end{array}$$

Subscript *l* ∈ {1,2, *r*} indicates decoding operation associated with encoders at MU1, MU2, and RN, respectively. MAP decoding is performed on the columns of Γ^ch^ with corresponding column from Γ_*r*_^*a*^ as* a priori* information. MAP decoding is performed using BCJR algorithm [17] to calculate the extrinsic LLR matrix Γ_*r*_^*e*^. Since Γ_*r*_^*e*^ is an (*L*_1_ + *L*_2_) × *mn* matrix consisting of alternate rows of packets from MU1 and MU2, they are isolated and deinterleaved to obtain *L*_1_^*a*^ and *L*_2_^*a*^. Each row in *L*_1_^*a*^ and *L*_2_^*a*^ constitutes the* a priori* LLRs of an RS codeword and can be decoded independently by applying ABP algorithm. After an iteration of ABP on all the rows, extrinsic information *L*_*ω*_^*e*^ and* a posteriori* information *L*_*ω*_^*p*^ for *ω* = 1,2 are obtained.

With *L*_*ω*_^*p*^, Berlekamp-Massey algorithm [18] is performed to decode the RS codes. The retrieved codes are represented as ${\overset{-}{S}}_{\omega }$. *L*_1_^*e*^ and *L*_2_^*e*^ are then interleaved and rows are combined into* a priori* LLR matrix Γ_*r*_^*a*^ for the next iteration of MAP decoder. The decoding terminates once all the RS codewords have been decoded or the maximum number of iterations is reached.

### 3.1. SISO Decoding of Convolutional Codes

One of the most popular MAP decoders used for decoding convolutional code is BCJR decoder. BCJR algorithm also finds application in iterative decoding of turbo code. We will briefly summarize the BCJR algorithm employed as network decoder in the proposed scheme. The BCJR decoder computes the* a posteriori* (APP) LLR *γ* ^*p*^(*c*_*i*_) for a bit *c*_*i*_ from the received RSCC codeword **y** = [*y*_1_, *y*_2_,…, *y*_*L*_] and* a priori* LLR *γ* ^*a*^, where *γ* ^*a*^(*c*_*i*_) = Γ_*r*_^*a*^(*i*, *j*), *i* = 1,…, *L*. The APP LLR is at the output of BCJR algorithm that is defined as

(5) $$\begin{array}{c} \gamma^{p}\left(\;c_{i}\;\right)=\log\left(\;\frac{P\left(c_{i}=+1\;|\;y\right)}{P\left(c_{i}=-1\;|\;y\right)}\;\right). \end{array}$$

BCJR decoder obtains as estimate of* a posteriori* LLR by incorporating the trellis of the code:

(6) $$\begin{array}{c} \gamma^{p}\left(\;c_{i}\;\right)=\log\left(\;\frac{\sum_{S^{+}}p\left(s_{i-1}=s^{\prime },s_{i}=s,y\right)/p\left(y\right)}{\sum_{S^{-}}p\left(s_{i-1}=s^{\prime },s_{i}=s,y\right)/p\left(y\right)}\;\right), \end{array}$$

where *S* is the set of all the states of trellis and *s*_*i*_ ∈ *S* is the state of the encoder at time *i*. *S* ^+^ is the set of ordered pairs (*s*′, *s*) corresponding to all state transitions (*s*_*i*−1_ = *s*′) → (*s*_*i*_ = *s*) caused by input *u*_*i*_ = +1, and *S* ^−^ is similarly defined for *u*_*i*_ = −1. Using Bayes' rule, (5) can be written as

(7) γpcilog⁡Py ∣ ci=+1Py ∣ ci=−1+log⁡Pci=+1Pci=−1=γeci+γaci,

where the second term *γ* ^*a*^(*c*_*i*_) represents* a priori* LLR and was applied as input to the decoder. The term *γ* ^*e*^(*c*_*i*_) = *γ* ^*p*^(*c*_*i*_) − *γ* ^*a*^(*c*_*i*_) is the extrinsic information that is obtained by subtracting* a priori* input from the output of the decoder and is passed onto the next decoder stage.

### 3.2. SISO Decoding of Reed-Solomon Codes

The next stage consists of ABP algorithm for SISO decoding of RS codes. ABP algorithm is a significant departure from the traditional hard decision decoding of RS codes. Besides being able to decode errors beyond maximum distance separable (MDS) capability, ABP algorithm enables iterative soft decision decoding in conjunction with other soft decision decoders and equalizers. Each iteration of the algorithm proceeds in two steps, updating the parity check matrix and calculation of extrinsic information.

We now briefly explain the ABP algorithm, details of which can be found in [17]. The extrinsic LLR matrix Γ_*ω*_^*e*^ of previous stage after deinterleaving becomes matrix *L*_*ω*_^*a*^ for this stage. For simplification of notations, let the LLR of bits in *i*th row at iteration *t* be denoted as

(8) $$\begin{array}{c} \lambda^{\left(t\right)}\left(\;c_{j}\;\right)=L_{\omega }^{a}\left(\;i,j\;\right),\;j=1,\ldots ,mn, \end{array}$$

where Λ^(*t*)^ = [*λ* ^(*t*)^(*c*_1_),…, *λ* ^(*t*)^(*c*_*j*_), …, *λ* ^(*t*)^(*c*_*mn*_)] acts as* a priori* information for an RS codeword. To update the parity check matrix *H*, |*λ* ^(*t*)^(*c*_*j*_)| is sorted in ascending order of magnitude and sorting index is stored as *π* = *j*_1_, *j*_2_, …, *j*_*mn*_. Columns of the matrix *H* are then reordered according to the permutation *π* to obtain *π*(*H*). Gaussian elimination then reduces the first independent *m*(*n* − *k*) columns to identity submatrix. Let this matrix be denoted as *H*′. Finally, inverse permutation is performed on the columns of *H*′ as follows:

(9) $$\begin{array}{c} H^{\prime \prime }=\pi^{-1}\left(\;H^{\prime }\;\right). \end{array}$$

Calculation of extrinsic information is performed using sum-product algorithm based on adapted parity check matrix *H*′′. For each bit of RS code, the extrinsic LLR is obtained as

(10) λetcj=∑i=1Hij′′=1mn−k2 tanh−1⁡∏p=1p≠j,Hip′′=1mntanh⁡λtcp2.

APP LLR is then updated as (11) to perform classical RS decoding:

(11) $$\begin{array}{c} \lambda^{\left(t+1\right)}\left(\;c_{p}\;\right)=\lambda^{\left(t\right)}\left(\;c_{p}\;\right)+\theta \cdot \lambda^{e\left(t\right)}\left(\;c_{j}\;\right), \end{array}$$

where 0 < *θ* ≤ 1 is a damping coefficient. Its value is taken to be 0.3 according to [19]. The extrinsic information *λ* ^*e*(*t*)^(*c*_*j*_) for the *i*th row obtained for this decoding iteration is saved into the matrix *L*_*ω*_^*e*^ as follows:

(12) $$\begin{array}{c} L_{\omega }^{e}\left(\;i,j\;\right)=\lambda^{e\left(t\right)}\left(\;c_{j}\;\right),\;j=1,\ldots ,mn. \end{array}$$

*L*_*ω*_^*e*^,   *ω* = 1,2, is then interleaved using Π_*ω*_ and rows of LLR are combined back into Γ_*r*_^*a*^. The combined LLR matrix Γ_*r*_^*a*^ is of size (*L*_1_ + *L*_2_) × *mn* and is of the form given in Figure 3. The iterations are run a predetermined number of times or until parity checks are satisfied.

### 3.3. Complexity Analysis

In this section, we analyze the complexity incurred by the proposed iterative decoding algorithm. Since encoding operation at the user nodes and encoding/decoding at relay are not different for both proposed and baseline schemes, we concentrate only on the decoding complexity in this paper. The following calculations are based on analysis done by Chen [15]. As discussed in earlier sections, the iterative decoder consists of two constituent decoders—BCJR and ABP algorithms. An iteration of BCJR or MAP decoding algorithm for RSCC requires *O*(12*Ω*(*L*_1_ + *L*_2_)) floating point operations, where *Ω* is the number of states of the convolutional coder. SISO decoding of RS code consists of three stages, which includes Gaussian elimination, belief propagation or sum-product algorithm, and RS hard decision decoding. Gaussian elimination process requires *O*(*mn*(*mn* − *km*)^2^) binary operations. If *θ* is the average row weight of adapted parity check matrix *H*′′, each iteration of sum-product algorithm requires *O*((*n* − *k*)*mθ* ^2^) floating point operations. Finally, performing RS hard decision decoding using Berlekamp-Massey algorithm requires *O*(*n* ^2^) finite field arithmetic operations [20].

With this information, we now analyze the approximate decoding complexity of the proposed algorithm and compare it with that of reference decoder. For each iteration of ABP algorithm, Gaussian elimination and belief propagation algorithm are run for maximum of *N*_iter_ number of times and after completion of iteration hard decision Berlekamp-Massey algorithm is applied. The iterations may terminate early for higher SNR values. Therefore, together with BCJR algorithm, the proposed iterative decoder requires at most

(13) ONiter12ΩL1+L2mn+n−kmθ2L1+L2

floating point operations. In the above equation, (*L*_1_ + *L*_2_) is the total number of RS codewords received from both of the mobile users. The number of binary operations required is

(14) $$\begin{array}{c} O\left(\;N_{iter}\left(\;L_{1}+L_{2}\;\right)mn{\left(\;mn-km\;\right)}^{2}\;\right). \end{array}$$

Since hard decision Berlekamp-Massey algorithm is applied after completion of *N*_iter_ number of sum-product algorithm iterations, there are at most

(15) $$\begin{array}{c} O\left(\;\left(\;L_{1}+L_{2}\;\right)n^{2}\;\right) \end{array}$$

finite field arithmetic operations.

If similar system is implemented with classical Viterbi algorithm for decoding RSCC, it will require *O*(2*Ω*(*L*_1_ + *L*_2_)) floating point operations. For Berlekamp-Massey algorithm, the number of finite field arithmetic operations required will be *O*((*L*_1_ + *L*_2_)*n* ^2^). It is obvious that complexity of the proposed algorithm is far greater than one-shot Viterbi-Berlekamp-Massey algorithm. This increased complexity is justified considering the significant improvement in bit error rate. Decoding complexity imposed by ABP algorithm can be further reduced by adopting newer algorithm for SISO decoding of RS codes; see [16]. Addressing this complexity issue will be the authors' future work.

## 4. EXIT Analysis

In this section we utilize extrinsic information transfer (EXIT) chart [18, 21] to analyze the iterative decoding behaviour of the proposed network-channel decoder. It allows us to visualize the decoding trajectory of iterative decoder components and prediction of BER performance. This in turn facilitates selection of suitable constituent codes for the scheme without time consuming Monte Carlo simulations for evaluating the performance of the scheme.

A fundamental assumption of EXIT chart analysis is that extrinsic information passed from one SISO decoder to another is a Gaussian distributed random variable. The LLR *λ*_*a*_ of* a priori* input for uncoded information *x* ∈ {+1, −1} is modelled as

(16) $$\begin{array}{c} \lambda_{a}=\mu_{a}x+n_{a}, \end{array}$$

where *n*_*a*_ is a Gaussian random variable with zero mean and variance *σ*_*a*_^2^. The variance must satisfy the condition *μ*_*a*_ = *σ*_*a*_^2^/2. The mutual information between *λ*_*a*_ and *x* is defined as

(17) Ix,λa12∑x=±1paξ ∣ x·log2⁡2paξ ∣ xpaξ ∣ x=−1paξ ∣ x=+1dξ,

where *p*_*a*_(*ξ*∣*x*) is conditional probability density function associated with* a priori* LLR *λ*_*a*_. Therefore, for* a priori* LLR *λ*_*a*_, mutual information is given as *I*_*a*_ = *I*(*x*, *λ*_*a*_). Similarly, mutual information for extrinsic output *λ*_*e*_ is obtained as *I*_*e*_ = *I*(*x*, *λ*_*e*_). To obtain EXIT chart, we artificially generate the* a priori* inputs *λ*_*a*_ to be fed into SISO decoder modules for given values of *I*_*a*_. Then the corresponding decoding algorithm of the block is invoked to produce extrinsic output *λ*_*e*_. The mutual information *I*_*e*_ is then evaluated using relation (16). Finally, EXIT chart is obtained as the graphical plot between *I*_*a*_ and *I*_*e*_. For decoding without any residual error, *I*_*e*_ should equal 1 for some value of *I*_*a*_.

**Figure 5:**
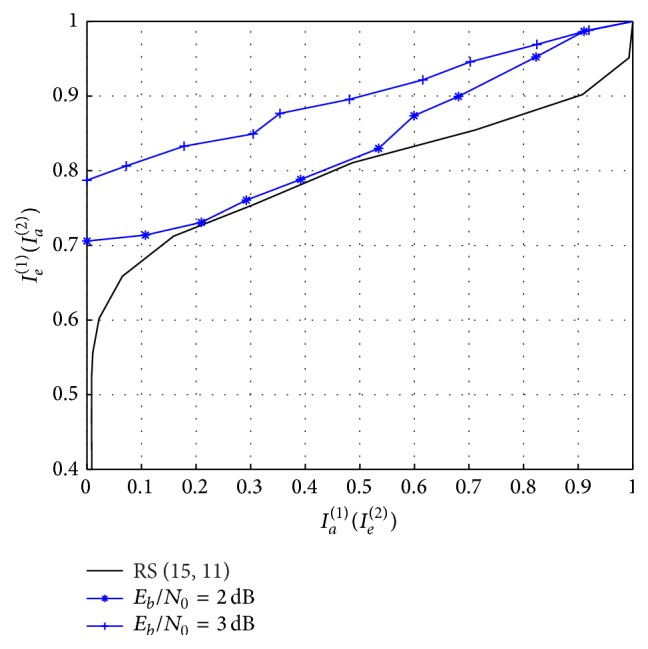
EXIT chart of the proposed network-channel decoder with (15, 11) RS code as channel code (outer code) and punctured (5, 7)_8_ RSCC acting as network code with code rate of 2/3.

**Figure 6:**
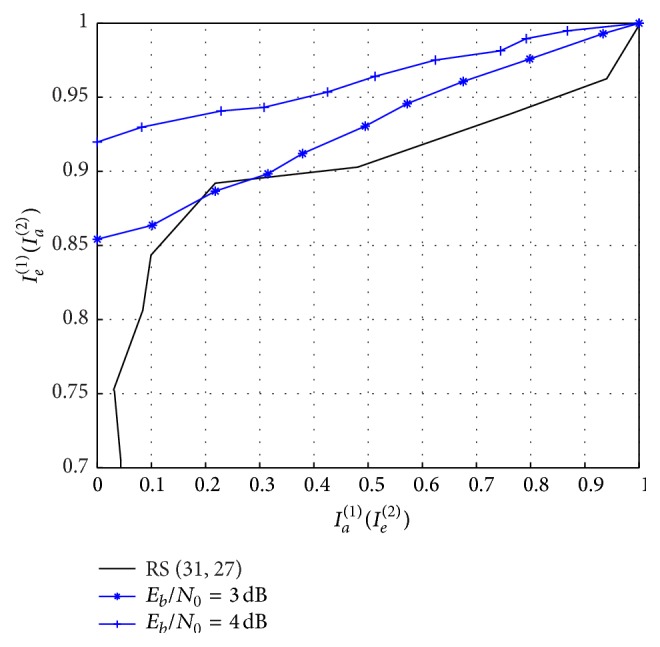
EXIT chart with (31, 27) RS code and punctured (15, 17)_8_ RSCC acting as network code with code rate of 2/3.

Figure 5 shows the EXIT characteristics of the proposed decoding scheme with (15, 11) RS code. The inner decoder (decoder 1 indicated as superscript in symbols *I*_*a*_^(1)^ and *I*_*e*_^(1)^) consists of punctured (5, 7)_8_ RSCC acting as network code with code rate of 2/3. The (*I*_*a*_^(1)^, *I*_*e*_^(1)^) curves are plotted with average channel *E*_*b*_/*N*_0_ of 2 dB and 3 dB. Inverse EXIT characteristics (*I*_*e*_^(2)^, *I*_*a*_^(2)^) are also obtained for decoder 2 (outer RS decoder consisting of ABP algorithm). It shows, at 2 dB, that the tunnel starts to open between EXIT curves of decoder 1 and decoder 2, and, at 3 dB, the tunnel is completely open. Therefore, the decoder bit error rate (BER) cliff is expected to start at 2 dB and can be verified in Figure 8. The EXIT chart for the system with (31, 27) RS code as channel code and punctured (15, 17)_8_ RSCC acting as network code is given in Figure 6, where turbo-cliff starts for *E*_*b*_/*N*_0_ ≥ 3 dB. The corresponding improvement in BER performance with decoding iterations is indicated in Figure 9.

**Figure 7:**
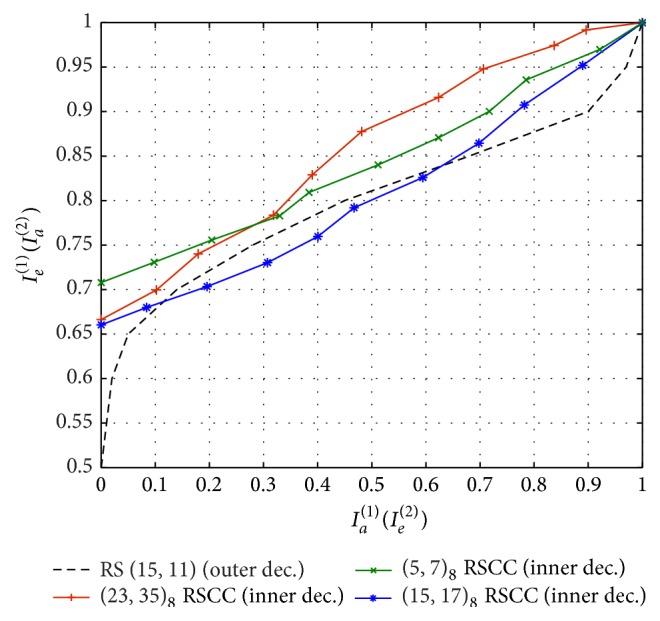
EXIT chart with (15, 11) RS outer code and different RSCC codes as network code.

EXIT chart can be used for selecting a suitable network code from the family of RSCC.  Figure 7 shows the EXIT chart of the proposed algorithm with RS (15, 11) as outer code (channel code) and different convolutional codes (network code). The three convolutional codes considered are with four-state (5, 7)_8_, eight-state (15, 17)_8_, and 16-state (23, 35)_8_ trellis. Similar to the earlier two cases, the channels are assumed to be Rayleigh flat fading with different links suffering an independent amount of fading. As can be seen in Figure 7, at *E*_*b*_/*N*_0_ = 2.5 dB, the tunnel between (*I*_*a*_^(1)^, *I*_*e*_^(1)^) and (*I*_*e*_^(2)^, *I*_*a*_^(2)^) curves is already opened for RSCC with trellis polynomials (23, 35)_8_ and (5, 7)_8_. With increase in channel *E*_*b*_/*N*_0_ values, the (*I*_*a*_^(1)^, *I*_*e*_^(1)^) curve moves upward for lower values of *I*_*a*_^(1)^ and the first opening in the tunnel appears for (5, 7)_8_ RSCC. The tunnel between the other two EXIT curves opens up subsequently with increase in SNR. Hence, coding scheme using (5, 7)_8_ convolutional code will encounter waterfall region earlier than the other two codes.

## 5. Simulation Results

In this section we demonstrate through simulations that proposed iterative network-channel decoder outperforms existing scheme. For comparison we consider the reference scheme proposed in [9]. The performance is evaluated for BPSK modulated signal transmitted over Rayleigh block fading channel; that is, the channel fading coefficient is assumed to be constant for the duration of one packet. As explained earlier, we consider MARC scheme with two users cooperatively transmitting to the BS. An intermediate relay node assists in the transmission through network coding. The SNR of MU-BS and RN-BS is assumed to be the same unless mentioned otherwise.

**Figure 8:**
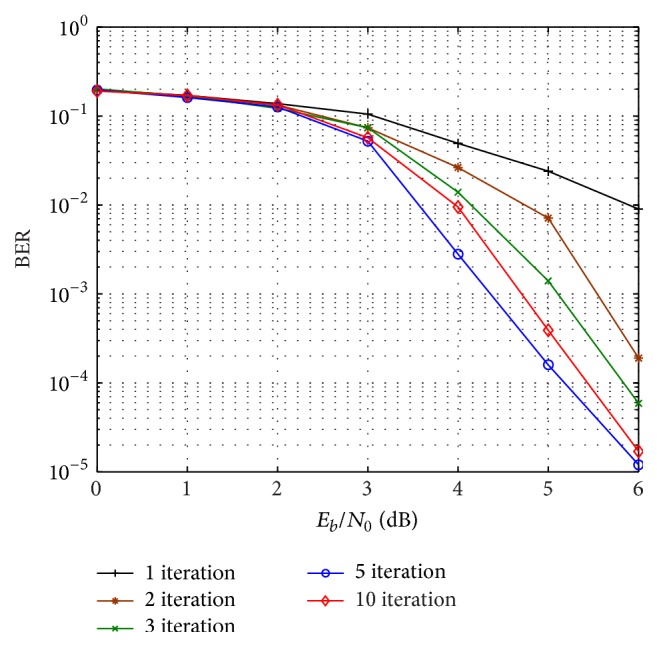
BER performance of the proposed scheme using (15, 11) RS code and punctured (5, 7)_8_ RSCC with number of decoding iterations over Rayleigh block fading channel.

**Figure 9:**
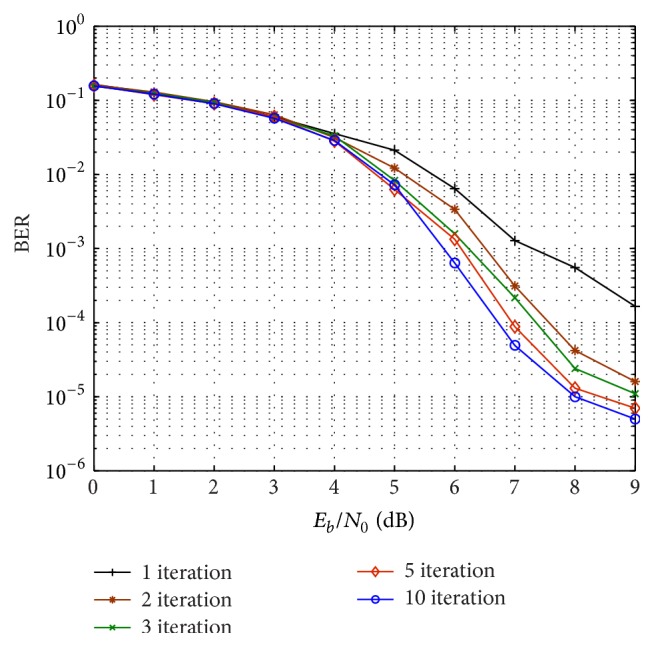
BER performance for (31, 27) RS code and (15, 17) RSCC combination with number of decoding iterations over Rayleigh block fading channel.

First we investigate the iterative convergence behaviour of the proposed design. Figure 8 shows BER performance of the proposed network-channel decoder with (15, 11) RS code as channel code and punctured (5, 7)_8_ RSCC as network code. It can be observed that iterative decoding gain is obtained for *E*_*b*_/*N*_0_ values higher than 2 dB, as predicted in EXIT chart of Figure 5. Error rate decreases with increase in iterations and there is no significant improvement in BER after 10 iterations. Figure 9 shows the performance of the iterative decoder with (31, 27) RS code and (15, 17) RSCC combination, in which iteration gain starts for *E*_*b*_/*N*_0_ ≥ 3 dB. The best BER performance is achieved for 10 iterations or more. This corresponds to EXIT chart in Figure 6, as explained earlier.

**Figure 10:**
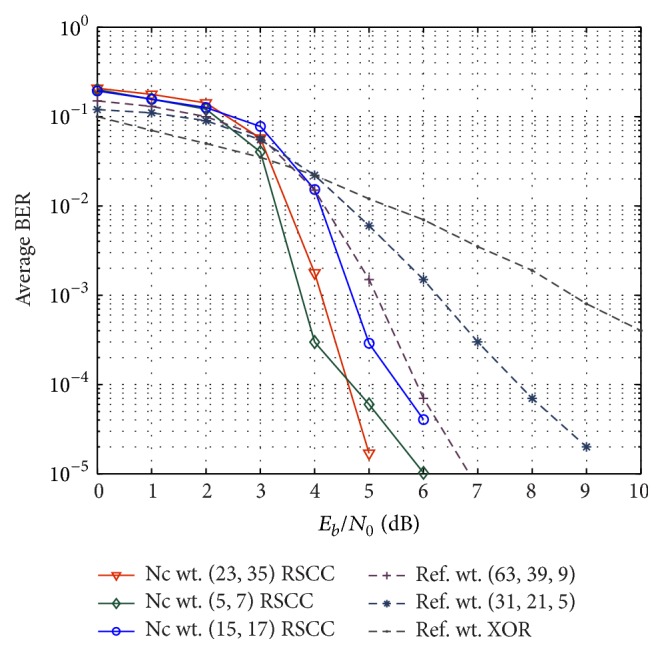
Performance comparison of the proposed iterative decoder with different reference schemes. Different network codes at relay node are evaluated for a fixed channel code at the user nodes.

Next we look at the interaction between channel and network code. For this purpose, we fix the channel code at the user nodes to (15, 11) RS codes and look at convolutional codes with different generator polynomials applied as network code. To compare the performance with XOR based network code and the reference scheme, the convolutional code at the relay is punctured to get rates close to 2/3. The proposed decoding algorithm is run for ten iterations. With all the links having the same average received SNR, Figure 10 shows average bit error rate of the MARC as function of *E*_*b*_/*N*_0_. For comparison we also show the results obtained in the reference scheme in [9] with a similar network-channel decoding scheme. It is observed that the average bit error probability of the proposed scheme has improved considerably compared to the baseline system. For instance, using punctured (23, 35)_8_ RSCC as network code, a gain of nearly 1.5 dB can be observed at a bit error rate of 10^−3^, as compared for (63, 39, 9) BCH code based network coding. Compared to (31, 21, 5) BCH code based network code, gain is even more significant (of 2.5 dB) at bit error rate of 10^−3^ whereas the gain of the proposed scheme with (5, 7)_8_ RSCC network code with respect to (63, 39, 9) and (31, 21, 5) BCH code based network code is 1 dB and 2 dB, respectively. For the purpose of comparison, Figure 10 also shows the performance of the baseline scheme with XOR based network code.

**Figure 11:**
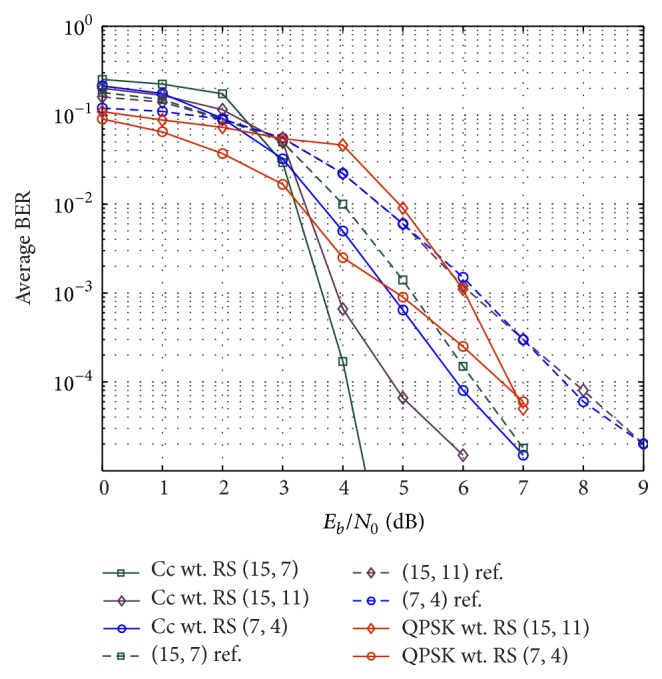
Performance comparison with different channel codes at the user nodes and fixed network coding at relay. Performance with QPSK modulated transmission is also shown in the figure.

Next we simulate another type of interaction between channel and network code where network coding at the relay node is kept fixed and channel code is varied.  Figure 11 illustrates the bit error rate of the proposed scheme with punctured (5, 7)_8_ RSCC as network code. The channel codes considered are (15, 7), (15, 11), and (7, 4) Reed-Solomon codes. It can be observed that relative gain in performance is higher than that of baseline scheme consisting of relatively complex (31, 21, 5) BCH code as network code, and channel codes being (15, 7), (15, 11), and (7, 4) BCH code. For example, to achieve bit error rate of 10^−4^, the relative gain of the proposed coding scheme with user channel code of (15, 7) RS code is about 2 dB better compared to corresponding reference scheme with (15, 7) BCH code. On the other hand the gain is 3 dB for (15, 11) block code used as channel code at the user nodes. But the gain is reduced to 2 dB for (7, 4) block codes used as channel code at the two user nodes. All the simulations performed in this paper use BPSK modulation. This can be easily extended to higher-order modulation. Two simulation curves which use QPSK modulation are also shown in Figure 11. The QPSK simulation is done for RS codes (15, 11) and (7, 4), but the network code remains the same. For other higher-order modulation designs, suitable combination of channel code and network code can be done using EXIT analysis discussed earlier.

**Figure 12:**
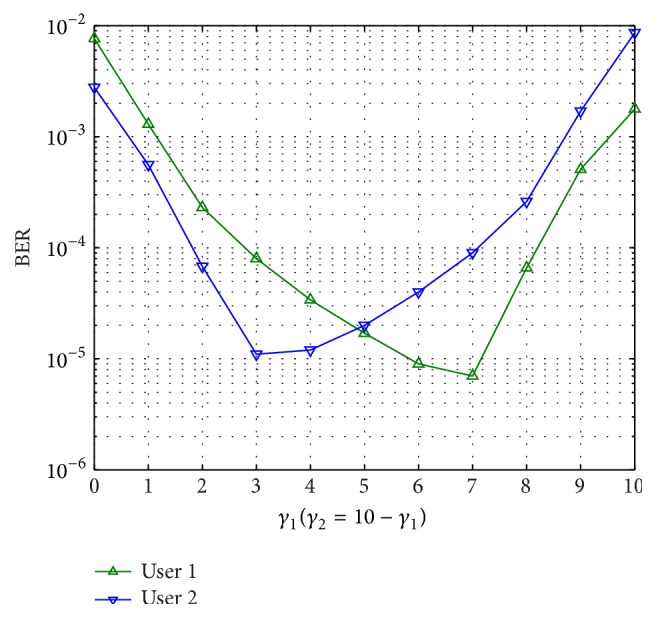
Performance of proposed scheme in asymmetric scenario over Rayleigh fading channel, with average relay-destination SNR of 8 dB. Average SNR of user 1 to destination is *γ*_1_ and user 2 to destination SNR is *γ*_2_ = 10 − *γ*_1_.

Figure 12 shows the performance of the proposed scheme in asymmetric SNR scenario. The information at both sources is encoded with (15, 7) RS code and network coding operation is done at the relay with (15, 17)_8_ RSCC as mother code. Similar to the scenario in [9], we study a case where the sum of SNRs for the direct links of the users is assumed constant; that is, *γ*_1_ + *γ*_2_ = 10 dB. Average received SNR of the relay-destination link is assumed constant with *γ*_3_ = 8 dB. It can be observed that, because of coupling between the two users at relay node, SNR of one link affects the BER performance of the other link also. When one of the users has bad link, both users will experience bad link performance regardless of the other user's link quality. Thus, the best performance is obtained with the proposed scheme when SNRs of both links are almost equal.

## 6. Conclusion

In this paper, we have discussed an improvement over the network-channel decoder for MARC setup. The received signal bits form a product code, where rows are formed by the individual packets of the two users ordered alternatively and columns are formed by network coding scheme used at the relay node. The proposed scheme consists of channel coding performed by Reed-Solomon codes at the user nodes and punctured convolutional code acting as network code at the common relay. Iterative soft decision decoding is performed at the receiver by exchanging extrinsic information between SISO decoders for RS and convolutional codes. Significant improvement in bit error rate performance is obtained with the proposed scheme compared to the baseline network-channel scheme. The EXIT analysis of the proposed decoder is presented to validate the decoding convergence results. Future works involve employment of lower complexity SISO decoder for RS codes and studying the properties of the decoder in network-channel coding scenario.

## Figures and Tables

**Table 1:** Transmission schedule at the user and relay nodes.

Time slot	Transmitter	Message	Receiver
1	MU1	*S*_1_ = *C* ^(1, *p*)^, *p* = 1,…, *L*_1_	RN, BS
2	MU2	*S*_2_ = *C* ^(2, *p*)^, *p* = 1,…, *L*_2_	RN, BS
3	RN	*S*_3_ = *P* ^(*r*, *p*)^, *p* = 1,…, *L*_*r*_	BS

## References

[1] Fitzek F. H. P., Katz M. D. (2006). *Cooperation in Wireless Networks: Principles and Applications*.

[2] Laneman J. N., Wornell G. W. (2003). Distributed space-time-coded protocols for exploiting cooperative diversity in wireless networks. *IEEE Transactions on Information Theory*.

[3] Zhao Y., Adve R., Lim T. J. Improving amplify-and-forward relay networks: optimal power allocation versus selection.

[4] Yang S., Belfiore J.-C. (2007). Towards the optimal amplify-and-forward cooperative diversity scheme. *IEEE Transactions on Information Theory*.

[5] Zhao B., Valenti M. C. (2003). Distributed turbo coded diversity for relay channel. *Electronics Letters*.

[6] Hunter T., Nosratinia A. (2006). Diversity through coded cooperation. *IEEE Transactions on Wireless Communications*.

[7] Zhou X., Cheng M., Anwar K., Matsumoto T. (2012). Distributed joint source-channel coding for relay systems exploiting source-relay correlation and source memory. *EURASIP Journal on Wireless Communications and Networking*.

[8] Chen Y., Kishore S., Li J. Wireless diversity through network coding.

[9] ur Rehman Ahsin T., Ben Slimane S. (2012). A joint channel-network coding based on product codes for the multiple-access relay channel. *ISRN Communications and Networking*.

[10] Menghwar G. D., Jalbani A. A., Memon M., Hyder M., Mecklenbräuker C. F. (2012). Cooperative space-time codes with network coding. *EURASIP Journal on Wireless Communications and Networking*.

[11] Menghwar G. D., Shah A. A., Mecklenbräuker C. F. Cooperative space-time codes with opportunistic network coding with increasing numbers of nodes.

[12] Du B., Zhang J. (2010). Parity-check network coding for multiple access relay channel in wireless sensor cooperative communications. *EURASIP Journal on Wireless Communications and Networking*.

[13] Fang W., Hu C., Sun Z., Hou S. Improved joint network-channel coding for the multiple-access relay channel.

[14] Zhou X., Lim A. O., Anwar K., Matsumoto T. Distributed joint source-channel-network coding exploiting source correlation for multiple access relay channel.

[15] Chen L. (2013). Iterative soft decoding of reed-solomon convolutional concatenated codes. *IEEE Transactions on Communications*.

[16] Bellorado J., Kavcic A., Marrow M., Li P. (2010). Low-complexity soft-decoding algorithms for Reed-Solomon codes—part II: soft-input soft-output iterative decoding. *IEEE Transactions on Information Theory*.

[17] Jiang J., Narayanan K. R. (2006). Iterative soft-input soft-output decoding of Reed-Solomon codes by adapting the parity-check matrix. *IEEE Transactions on Information Theory*.

[18] Brink S. T. (2001). Convergence behavior of iteratively decoded parallel concatenated codes. *IEEE Transactions on Communications*.

[19] Lin S., Costello D. J. (2004). *Error Control Coding*.

[20] Garrammone G. (2013). On decoding complexity of reed-solomon codes on the packet erasure channel. *IEEE Communications Letters*.

[21] Hanzo L. L., Maunder R. G., Wang J., Yang L.-L. (2011). *Near-Capacity Variable-Length Coding: Regular and EXIT-Chart-Aided Irregular Designs*.

